# Protein contact network explorer: topological analysis of protein structures

**DOI:** 10.3389/fbinf.2026.1870542

**Published:** 2026-07-07

**Authors:** Akhurath Ganapathy, Sanjana Vijay Krishnan, Arnold Emerson Isaac

**Affiliations:** Bioinformatics Programming Laboratory, Department of Bioscience, School of Bio Science and Technology, Vellore Institute of Technology, Vellore, Tamil Nadu, India

**Keywords:** graph-theoretic measures, network analysis, protein contact networks, protein stability, structural biology

## Abstract

**Introduction:**

The functions of proteins are primarily governed by coordinated interactions among amino acid residues throughout their three-dimensional structures. Large-scale determination of protein structures has long been made possible by experimental and computational methods; however, studying complex, dynamic, or multimeric systems remains challenging. Protein contact networks (PCNs) offer a graph-based representation of residue-level interactions and enable the application of network analysis techniques to structural data. Nevertheless, many existing tools mainly focus on creating static networks, which limits analytical flexibility.

**Methods:**

In this study, we introduce Protein Contact Network Explorer (PCNE), a tool for simple construction, visualisation, and analysis of protein contact networks derived from structure data.

**Results:**

The tool provides flexible residue contact definitions, the exploration of interactive networks, and the extraction of graph-theoretic measures relevant to understanding protein stability, allosteric communication, and functional organisation.

**Discussion:**

PCNE supports the analysis of key interaction patterns, facilitating both exploratory and hypothesis-driven research in structural biology. The PCNE can be accessed via https://lactdr5rfibhg9m5tmamwg.streamlit.app/.

## Introduction

### Importance of protein structure

Protein structures determine how proteins function, interact, and respond to their environment, making them a vital aspect of cellular and molecular biology. A protein’s significance depends on its binding partners, catalytic activity, stability, and regulatory behaviour, all of which are shaped by its three-dimensional conformation. Over time, advancements in low-throughput experimental methods for determining protein structures have paved the way for higher-resolution techniques such as X-ray crystallography, nuclear magnetic resonance (NMR) spectroscopy, and cryo-electron microscopy (cryo-EM). The application of these techniques, along with newer artificial intelligence-driven methods, most notably AlphaFold2 (Senior et al., 2020), has allowed for a more detailed visualisation of macromolecular structures and simplified the process of predicting structures directly from amino acid sequences (Liu et al., 2020; Wuyun et al., 2024; Qin et al., 2024). Protein structures are commonly characterised by primary, secondary, tertiary, and quaternary levels, reflecting their hierarchical organisation. Local interactions, like hydrogen bonding, form secondary structural elements, while higher-order folding and subunit assembly define functional domains and cooperative behaviour (Siciliano et al., 2024; Motlagh et al., 2014).

The analysis of protein structures today is increasingly supported by computational approaches that complement experimental advances, with recent deep learning models performing well in predicting secondary and tertiary structures across various protein families (Meng et al., 2025; Jänes and Beltrao, 2024). Proteins, once thought to be rigid, static entities, are now recognised as dynamic systems with multiple conformational states and transitions between them (Nussinov et al., 2023). The formation of toxic oligomers and amyloid fibrils, caused by protein misfolding and aggregation, further emphasises the significance of a protein’s structural organisation (Knowles et al., 2014). This abnormal folding has been associated with several neurodegenerative diseases such as Alzheimer’s and Parkinson’s disease (Knowles et al., 2014; Rinauro et al., 2024; Koszła and Sołek, 2024). The importance of analytical frameworks over static structural descriptions is emphasised here to better understand internal interaction patterns within proteins, highlighting the increasing need for analytical approaches to interpret proteins—particularly those with large, complex structural datasets, transient conformational states, and large macromolecular assemblies.

### Importance of protein contact networks

The study of PCNs has gained significant importance in structural bioinformatics, as it transforms three-dimensional protein structures into graph-network models (Di Paola, 2014). In these models, amino acid residues are represented as nodes, while their interactions are depicted by edges. This mathematical analogy is vital because it helps connect static structural data with dynamic biological functions (Chak et al., 2016). Analysing folding dynamics and structural stability requires the use of PCNs. Researchers can predict folding rates and identify critical pathways that guide a protein from its unfolded form to its native conformation by calculating topological invariants such as contact order (Guzzi et al., 2022). Furthermore, PCN analysis serves as an effective approach for functional characterisation, particularly for understanding how allosteric regulation operates. Researchers can pinpoint the exact residue-level “hotspots” responsible for signal transmission throughout the molecule by detecting “hubs” (highly connected residues) and modular substructures (Piovesan et al., 2016; Aydınkal et al., 2019).

### Related work

Protein contact network analysis has become a valuable method for exploring protein functions at the structural level, and researchers have developed numerous computational tools to study PCNs. Among the most widely used is RING (Residue Interaction Network Generator), which constructs interaction networks from protein structures by detecting non-covalent interactions such as hydrogen bonds, salt bridges, and van der Waals contacts (Doncheva et al., 2011). Building on this foundation, PCN Miner offers a comprehensive range of network analysis capabilities, enabling the calculation of betweenness centrality, clustering coefficients, and other key topological measures that help identify crucial residues (Guzzi et al., 2022). NAPS (Network Analysis of Protein Structures) adopts an alternative approach by focusing on comparative analysis of different protein structures, which is useful for examining conformational changes and allosteric regulation (Chak et al., 2016). Other tools, like RINAnalyzer, provide versatility through customisable settings for defining residue interactions (Piovesan et al., 2016). PyInteraph introduces network analysis into molecular dynamics simulations through its Python-based framework (Tiberti et al., 2014).

Data on protein structures has grown quickly due to improvements in experimental techniques and structure prediction methods; however, extracting meaningful insights from these structures remains challenging. Static three-dimensional representations offer useful spatial information about protein structures, but they do not show interaction patterns, residue connectivity, or key regions within proteins. To facilitate intuitive exploration of residue-level interactions, more user-friendly analytical tools need to be created, keeping users’ computational backgrounds in mind.

In this work, we introduce an interactive web-based tool for constructing PCNs and analysing them directly from Protein Data Bank (PDB) structures. The tool supports both X-ray crystallographic structures and NMR ensembles, enabling users to select specific chains or models for analysis. Protein structures are represented using a coarse-grained alpha-carbon framework, where residues serve as nodes and network connectivity is based on spatial proximity. The tool also calculates node degree, clustering coefficient, closeness centrality, and betweenness centrality, allowing for quantitative assessment of residue connectivity. It integrates interactive three-dimensional visualisation with filtering options to facilitate exploratory analysis. Users can highlight hub residues, residues of particular biochemical classes, and nodes with high centrality values, supporting a more targeted study of structural features and interaction patterns. Additional options, such as degree-based filtering and contact frequency distributions, enable more comparative and descriptive analyses. User-friendliness and interpretability are maintained through its implementation as a browser-based application, making the analysis of protein contact networks more accessible for both educational and research purposes.

## Methods

**FIGURE 1:**
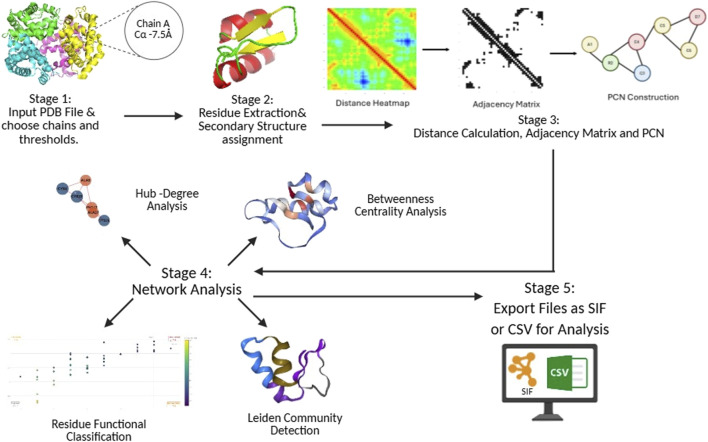
Workflow of PCNE illustrating the major stages of analysis.

PCNE provides a framework for the construction, visualization and analysis of residue-level protein contact networks from protein structures. The platform integrates network generation, topological analysis, community detection, structural mapping and network export functionalities within a single interactive environment as illustrated by Figure 1.

### Input data and structure parsing

The tool is designed to accept protein structural information in PDB format files generated from X-ray crystallography and NMR spectroscopy. For structures derived from X-ray crystallography, users can select a specific polypeptide chain; for NMR-determined structures, the tool supports multi-model ensembles and permits the selection of an individual structural model. Atomic coordinates corresponding to the selected residue representation were extracted from the chosen model or chain for subsequent analysis. These representations provide different degrees of structural abstraction, while maintaining the biologically relevant information important for network analysis.

### Protein contact network construction

#### Node representation

**FIGURE 2:**
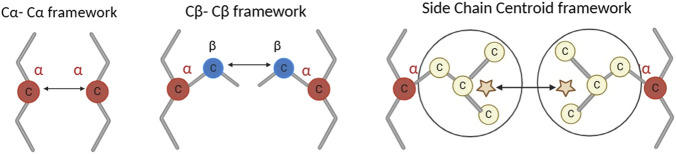
Comparison of residue representation frameworks used for PCN construction. Residues can be represented using Cα atoms, Cβ atoms, or side-chain centroids calculated based on non-hydrogen atoms. The star symbol represents the computed side-chain centroid.

The tool supports multiple residue representations for protein network construction, allowing users to define nodes using Cα, Cβ, or side-chain centroids (excluding hydrogen atoms), as shown in Figure 2. Cα atoms provide a coarse-grained representation of the protein backbone and are commonly used for global topological analysis. Cβ-based representations yield better networks because they incorporate additional information, such as side-chain orientation. Side-chain centroid representations provide an estimated measure of residue interaction positioning. From the chosen node representation, the distance between all pairs of nodes is calculated for further analysis.

#### Distance calculation

For two residues i and j with the Cartesian co-ordinates ( $x_{i},y_{i},z_{i}$ ) and ( $x_{j},y_{j},z_{j}$ ), the Euclidean distance is computed as

$$d\left(i,j\right)=\sqrt{{\left(x_{i}-x_{j}\right)}^{2}+{\left(y_{i}-y_{j}\right)}^{2}+{\left(z_{i}-z_{j}\right)}^{2}}$$

The computed values are stored in an NxN distance matrix (where N is the number of residues), with each distance value rounded to 4 decimal places to ensure numerical clarity without losing spatial information.

#### Adjacency matrix definition

A binary adjacency matrix is generated using a user-defined threshold distance, *rc*.

$$A\left(i,j\right)=\left\{\begin{array}{l} 1,d\left(i,j\right)\leq r_{c}.\\ 0,d\left(i,j\right)>r_{c}. \end{array}\right.$$

Where A (i, j) indicates the presence (1) or absence (0) of an edge, and d (i,j) denotes the distance between the two nodes considered. This binary matrix forms the basis for the PCN, where an edge signifies spatial proximity between residues.

#### Network metrics and topological analysis

Following network construction, several graph-based metrics are utilised to analyse residue connectivity and their functions within the protein structure.

##### Node degree and hub identification

The degree $k_{i}$ of a residue i, represents the number of residues it is in contact with

$$k_{i}=\sum_{j}A_{ij}$$

Where $A_{ij}$ is an element of the adjacency matrix. Residues with degrees in the top percentile (e.g., top 10%) are identified as hub residues, which often correspond to structurally or functionally significant regions.

##### Local clustering coefficient

The local clustering coefficient measures the tendency of a residue’s neighbours to form interconnected clusters. For a residue i with degree $k_{i}$ –

$$C_{i}=\frac{2e_{i}}{k_{i}\left(k_{i}-1\right)}$$

where $e_{i}$ is the number of edges between the neighbours of residue i. This metric portrays the local cohesiveness within the protein structure.

##### Closeness centrality (normalised)

This metric quantifies the closeness of a particular residue with all the other residues in the network. The tool is made to incorporate the Wasserman and Faust normalization, to ensure values between 0 and one even for disconnected graphs.

$$C_{close}\left(i\right)=\frac{n-1}{\sum_{j\neq i}d\left(i,j\right)}\;.\;\frac{n-1}{N-1}$$

Where.

- d (i,j) is the shortest path distance
- n is the number of reachable nodes from residue i
- N is the total number of residues

This measure proves essential for identifying residues central to global communication within the protein.

##### Betweenness centrality

Betweenness Centrality quantifies a node’s influence on information flow through the network. It is defined as

$$C_{between}\left(i\right)=\sum_{s\neq i\neq t}\frac{\sigma_{st}\left(i\right)}{\sigma_{st}}$$

Where.

-
$\sigma_{st}$ is the total number of shortest path lengths between residues s and t
-
$\sigma_{st}\left(i\right)$ is the number of those paths that pass through residue i

Residues with high Betweenness centrality often act as structural or functional bottlenecks.

##### Community detection

A Community, Module, or Cluster can be defined as a group of residues in a protein that are densely connected by the formation of tightly interacting regions, local structural modules, and functional groups or domains.

The tool employs the Leiden Community Detection Algorithm to detect which residues in the protein network interact more strongly with one another than the rest of the protein, and display the modularity score, which is defined as-

$$Q=\frac{1}{2m}\sum_{ij}\left[A_{ij}-\frac{k_{i}k_{j}}{2m}\right]\;\delta \left(c_{i},c_{j}\right)$$

Where.

- Q is the modularity score, a measure of the strength of division of the network into communities
-
$A_{ij}$ is the adjacency matrix element
-
$k_{i}$ and $k_{j}$ are the degrees of residues i and j
- m is the total number of edges in the network
-
$c_{i},c_{j}$ are the community assignments of residues i and j
-
$\delta \left(c_{i},c_{j}\right)$ is the Kronecker-Delta function,

$$\delta \left(c_{i},c_{j}\right)=\left\{\begin{array}{l} 1\;if\;c_{i}=c_{j}\\ 0\;otherwise \end{array}\right.$$

#### Residue classification and visual encoding

Residues are categorised into biochemical groups–hydrophobic, polar, positively charged, and negatively charged–which are represented by different node colours in the network visualisation, making interpretation straightforward. Node sizes are also scaled proportionally to the node’s degree, helping to visually emphasise highly connected nodes.

### Interactive filtering and visualisation

Table 1 presents the interface, which offers multiple filtering modes to enable targeted visualisation of protein contact networks.

**TABLE 1:** Interactive filtering and visualisation modes.

Mode	Description
a) Show all	Default representation; displays all residues and their interactions
b) Hub residue view	Highlights residues with high connectivity (high degree), enabling quick identification of structurally or functionally important residues
c) Hydrophobic core view	Displays only hydrophobic residues, allowing visualization of the protein’s core and internal packing
d) Closeness centrality view	Shows residues with high normalized closeness centrality—those topologically close to most others and potentially involved in global communication
e) Shortest path (betweenness) view	Emphasizes residues with high betweenness centrality, identifying bottleneck residues mediating information flow
f) Degree viewer	Allows inspection of residue connectivity by degree, supporting analysis of local contact density and neighborhood size

Additionally, an option to hide unmatched nodes allows for cleaner visualisation by removing residues that do not meet the selected filtering criteria. A colour legend toggle is provided to preserve interpretability when residue–type–based colouring is enabled.

The interface also supports the simultaneous visualisation of detected communities within both the protein contact network and the three-dimensional protein structure. Community membership can also be analysed using multiple structural rendering modes, such as cartoon, backbone trace, ball-and-stick, and surface-mesh representations. Distinct colours have also been used to differentiate the communities across both the network and structural views. In addition to this structural visualisation, a residue-community summary list is provided, displaying the cluster-wise residue composition and the distribution of secondary structure elements, such as helices, beta sheets, and loop regions.

In addition to community-based analysis, the tool also includes an interactive degree-betweenness scatter plot for residue-level topological assessment. The relationship between the residue connectivity and importance within the PCN is highlighted by the scatter plot. Residues are categorized into groups such as global critical hubs, structural hubs, bottlenecks and peripheral hubs (Table 2).

**TABLE 2:** Definitions of residue categories identified through degree-betweenness analysis in PCNE.

Category	Degree	Betweenness	Functional interpretation
Global critical	High	High	Residues combining strong local connectivity with high communication centrality, occupying biologically important positions within the network
Structural hubs	High	Low	Highly connected residues with critical importance in network stability and local organization of the protein structure
Bottleneck	Low	High	Residues that act as bridges between different regions of the protein, involved in long-range communication and allosteric mediation
Peripheral	Low	Low	Residues with limited influence on the structure, typically located near the outer regions of the protein network

Structural inference is further facilitated by simultaneously mapping these residue categories onto the protein’s three-dimensional structure, using the same rendering modes as in community analysis.

### Software implementation

The tool was developed in Python and deployed as an interactive web-based application using Streamlit. This offered a lightweight framework for building the user interface and enabled real-time interaction with protein structures and contact visualisations. Protein structure parsing and extraction of residue-level information were carried out using Biopython, facilitating efficient handling of PDB files, chain and model information, and parameter selection. NumPy and SciPy were employed for core numerical computations, such as calculating inter-residue distances and generating adjacency matrices.

Data organisation, filtering, and result storage were managed effectively using Pandas, which facilitated handling the network and residue-level features. Protein contact networks were constructed and analysed with NetworkX, with residues represented as nodes and inter-residue interactions as edges. Community detection analysis was implemented using igraph and the Leidenalg package, enabling the identification of communities of residues within protein contact networks.

Plotly and Matplotlib were used for interactive visualisation of network structures, three-dimensional representations of protein and continuous colour generation for NGL-based structural heatmaps. The processing and rendering of structural representations within the interface were supported by Pillow, while PyDSSP was used for secondary-structure analysis of helices, beta strands, and loop regions in the protein structure. All components were integrated in a way that the user-friendly web application ensured effective data processing, interactive analysis, and reproducible results.

## Results

### Case study protein selection

Cytochrome c (PDB ID: 1J3S) was selected as the protein of interest for this case study because it features a well-understood, compact, globular, single-domain topology. Its clearly defined tertiary structure makes it an ideal candidate for residue-level PCN analysis. To focus solely on the protein, the network-building process was restricted to the polypeptide backbone, excluding all HETATM entries and non-protein ligands. For cytochrome c, this involved removing the heme iron core. By considering only residue-residue contacts, the resulting distance graph and all subsequent topological properties, such as degree and betweenness centrality, reflect the intrinsic structural framework and evolutionary folding patterns of the protein. Residue-residue contacts were identified using a distance cutoff (rc) of 7.5 Å, applied uniformly across the entire structure to generate the adjacency and distance matrices, which were used in all subsequent analyses.

### PCN construction and nodal network visualization

**FIGURE 3:**
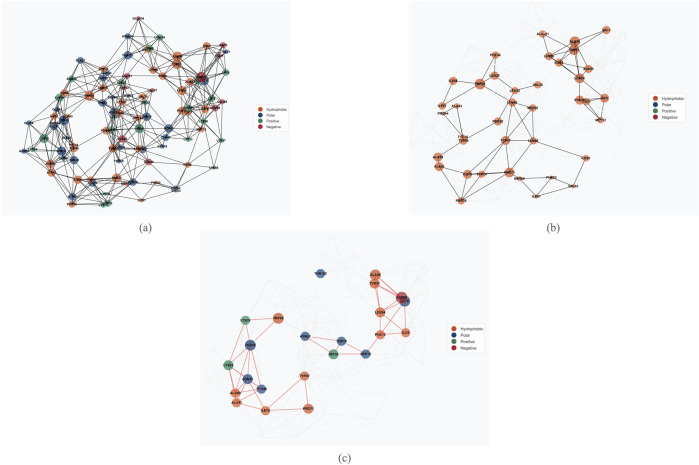
PCN analysis of Cytochrome c. **(a)** Full-network representation at 7.5 **(b)** Isolated Hydrophobic-core scaffold; **(c)** High-degree hub residues and their communities.

The residue-level PCN of cytochrome c was generated using the Cα atoms with a distance cutoff of 7.5 Å (Figure 3a), creating a connected graph that captures the spatial arrangement of inter-residue contacts. While the tool supports network construction using Cα atoms, Cβ atoms or side-chain centroids as node representations, the Cα framework was chosen for the case study owing to its widespread use in coarse-grained network topology analysis. This full-network representation forms the baseline topology from which targeted subnetworks are derived. To explore the contribution of the protein’s structural core, the PCN was selectively filtered to retain only hydrophobic residues (Figure 3b). This hydrophobic-core subnetwork isolates the densely connected internal scaffold of the protein, emphasising residues that mainly contribute to structural stability rather than surface interactions. Additionally, a hub-focused projection of the PCN was produced by selecting residues in the top 10% of the degree distribution (≥90th percentile) (Figure 3c). These hubs represent highly connected residues, identified relative to the degree distribution of the network and are highlighted alongside their identified communities, with edges shown in orange. Specific hub residues can also be targeted using the focus feature.

### Distance and adjacency plots

**FIGURE 4:**
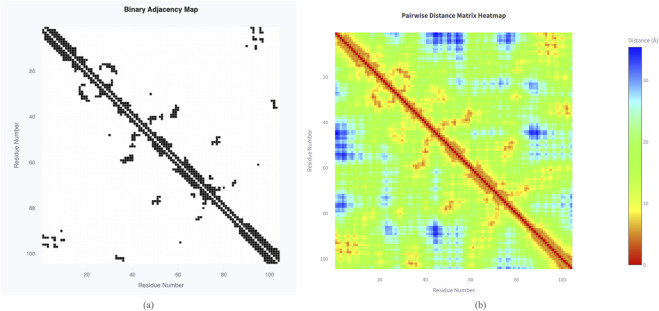
Matrix-level topological analysis. **(a)** Contact map showing the N-C terminal clamping; **(b)** Distance distribution highlighting the compact globular architecture.

The global topological structure of Cytochrome c is represented by the Adjacency and Distance matrices, which serve as a blueprint of the overall tertiary organization of the protein. The clusters in the top-right and bottom-left corners of the adjacency map (Figure 4a) reveal that there is spatial proximity between the residues located near the N- and C-terminal regions, despite being positioned at opposite ends of the protein chain. Such long-range interactions are consistent with the compact folded structure of the protein surrounding the central heme group.

The distance heatmap (Figure 4b) represents pairwise inter-residue Euclidean distance values, where the colour intensity corresponds to the spatial separation between residues. The heatmap displays a relatively narrow and uniform colour gradient without significant high-distance “hot spots”, suggesting that the protein adopts a compact, globular organization. The absence of isolated, high-distance regions (red patches) away from the diagonal further indicates limited extended or highly flexible terminal regions, consistent with the structural stability expected for an efficient electron carrier protein. The top-right and bottom-left regions tend to show patches of high-distance values due to interactions between residues positioned near opposite ends of the protein sequence, reflecting long-range tertiary contacts within the protein fold. The compact interaction distribution seen in the heatmap is also reflected in the relatively high residue connectivity and clustering observed in the PCN.

#### Network analysis

The topological analysis of the Cytochrome c contact network revealed a structure comprising 104 nodes and 409 edges. The resulting graph density was 0.0764, indicating a relatively sparse yet highly specific architecture typical of globular proteins. To identify the most structurally important residues, a hub analysis was conducted using a 90th percentile threshold (top 10% of the degree distribution). This criterion identified 23 hub residues with an average degree of 10.7. These hubs form the primary anchors of the protein, supporting long-range communication, structural stability, or both. The detailed topological metrics for these hubs are summarised in Table 3.

**TABLE 3:** Topological metrics for identified hub residues (Top 10% by Degree).

Residue	Type	Degree	Clust. coeff	Closeness	Betweenness
ASP-93	Negative	13	0.436	0.268	0.041
GLY-6	Polar	12	0.5	0.247	0.022
THR-40	Polar	12	0.424	0.307	0.079
TRP-59	Hydrophobic	12	0.424	0.33	0.142
ALA-96	Hydrophobic	12	0.47	0.27	0.032
HIS-18	Positive	11	0.473	0.279	0.051
ASN-52	Polar	11	0.491	0.268	0.027
LYS-53	Positive	11	0.509	0.266	0.021
PRO-71	Hydrophobic	11	0.455	0.254	0.043
LEU-94	Hydrophobic	11	0.455	0.281	0.059
TYR-97	Hydrophobic	11	0.473	0.273	0.038
ILE-9	Hydrophobic	10	0.467	0.253	0.043
PHE-10	Hydrophobic	10	0.533	0.261	0.061
SER-15	Polar	10	0.467	0.263	0.068
THR-19	Polar	10	0.4	0.261	0.023
ASN-31	Polar	10	0.422	0.272	0.043
LYS-39	Positive	10	0.467	0.301	0.055
THR-49	Polar	10	0.511	0.284	0.08
ALA-50	Hydrophobic	10	0.533	0.278	0.036
ALA-51	Hydrophobic	10	0.511	0.252	0.02
TYR-67	Hydrophobic	10	0.511	0.267	0.04
ILE-75	Hydrophobic	10	0.511	0.249	0.03
THR-102	Polar	10	0.467	0.29	0.056

### Community detection and structural mapping

**FIGURE 5:**
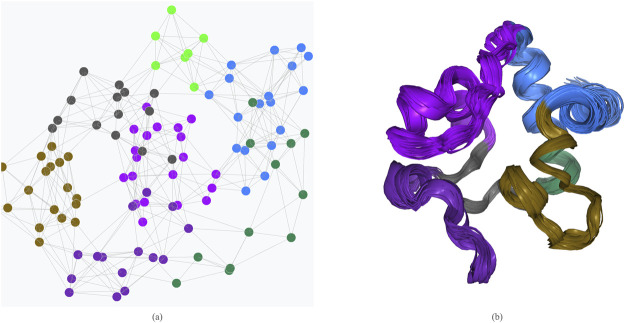
Community detection analysis. **(a)** PCN coloured according to Leiden algorithm community assignments; **(b)** 3-dimensional structural representation of cytochrome c coloured by cluster membership.

Community detection using the Leiden algorithm identified seven distinct communities within the protein contact network of cytochrome c, giving a modularity score of 0.6503. This indicates the presence of well-defined groups of residues which are densely connected within their community. The detected communities were visualized with both the protein contact network (Figure 5a) and the three-dimensional structure of the protein (Figure 5b). Different communities were represented using distinct colours, enabling the analysis of topological and spatial distributions.

Analysis of the composition of these communities yielded distinct distributions of secondary structural elements across the identified clusters. While some communities were predominantly made up of alpha helices (e.g., Cluster 2 with 89% helix), others were rich in looped regions (e.g., Cluster 1 with 100% loop). Some additional communities constituted mixed structural compositions. These observations pointed out that the detected communities were able to capture structurally coherent regions of the protein and a modular organsation of the residue interactions within the folded protein.

#### Degree-betweenness analysis

**FIGURE 6:**
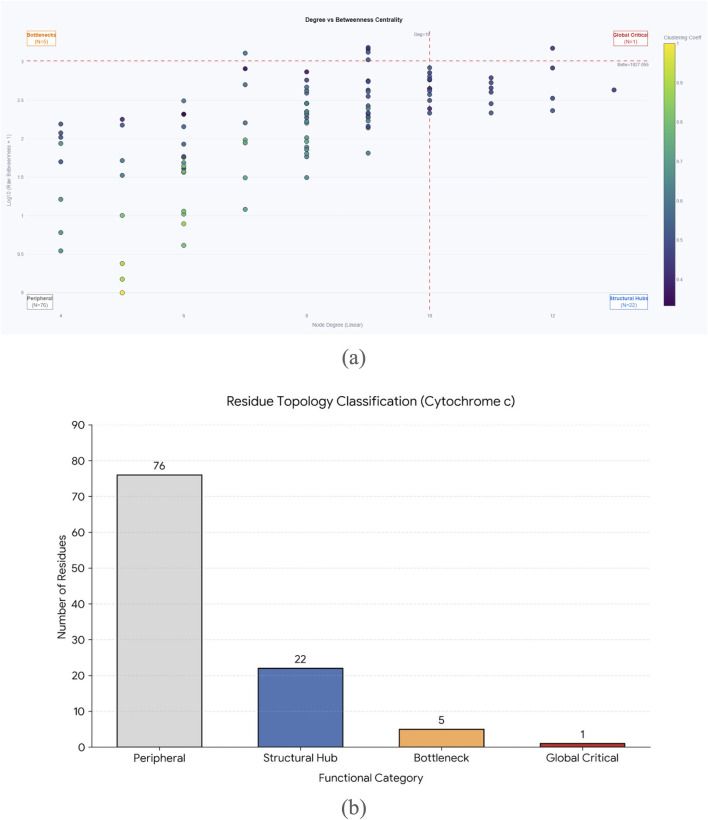
Functional classification of residues. **(a)** Scatter plot mapping residue roles based on connectivity and traffic; **(b)** Distribution chart showing the number of residues assigned to each functional role.

The relationship between nodal degree and betweenness centrality was analysed to classify residues based on their specific topological roles within the Cytochrome c network (Figures 6a,b). For all these calculations, the degree threshold was set at the 90th percentile, while the betweenness threshold was set at the 95th percentile. These thresholds are indicated by the vertical and horizontal dotted lines in Figure 6a. Peripheral residues (n = 76) are nodes with both low degree and low betweenness, typically found on the protein surface or within flexible loops. They have minimal influence on the overall stability of the network.

Structural Hubs (n = 22): Characterised by a high degree but low betweenness (e.g., GLY-6, HIS-18), these residues are the main pillars of the hydrophobic core. They form a dense network of local contacts that provide the mechanical rigidity necessary to maintain the protein’s globular shape.

**FIGURE 7:**
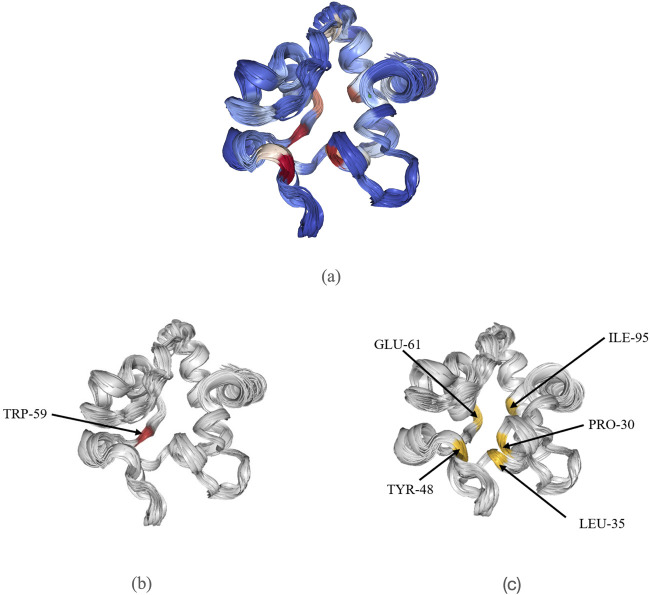
3D visualization of residue categories identified through degree-betweenness centrality. Colours represent the topological classification of residues within the PCN. **(a)** Betweenness Centrality. **(b)** Global Critical Hubs. **(c)** Bottlenecks.

Bottlenecks (n = 5): These residues have a low degree but high betweenness centrality (PRO-30, LEU-34, TYR-48, GLU-61, ILE-95) (Figure 7c). They serve as vital bridges connecting different structural modules. Their limited connectivity suggests a potential role in mediating communication between weakly connected regions of the network (makes them vulnerable points where a single mutation could potentially isolate entire sections of the network).

Global Critical Hub (n = 1): The residue TRP-59 (Figure 7b) is the most structurally important node. Acting as both a highly connected anchor and a central communication relay, it coordinates the integration of secondary structure elements and has a biologically essential topological position, combining high connectivity with strong communication centrality within the protein network. It is also essential for the rapid signal transfer needed for redox function.

The identified residue categories were further mapped onto the 3-dimensional structure of the protein based on their betweenness centrality, facilitating the structural interpretation of their topological properties (Figure 7). The different categories, coloured differently, can be easily compared in terms of network topology and protein structure. The spatial distribution of these classes of residues shows that topologically crucial residues are dispersed throughout the folded protein, instead of being isolated in a single structural element. This is consistent with the compact and interconnected structure seen in Cytochrome c, where the protein’s structural stability and communication pathways are owed to interactions spanning across the protein structure.

#### SIF cytoscape integration

To enable advanced visualization, the Cytochrome c contact network was exported in a Simple Interaction File (SIF) format. This format describes the interactome as a series of triplets: Source Node, Interaction Type, and Target Node. The resulting SIF file comprises 409 unique residue-residue (pp) interactions. It served as a key input for Cytoscape, facilitating the mapping and visualisation of the protein contact network. A sample of the interaction entries is shown in Table 4.

**TABLE 4:** Representative snippet of the exported SIF.

Source residue	Interaction	Target residue
GLY-1	pp	ASP-2
GLY-1	pp	VAL-3
GLY-1	pp	ALA-92
GLY-1	pp	ASP-93
GLY-1	pp	ALA-96
ASP-2	pp	VAL-3
ASP-2	pp	GLU-4
ASP-2	pp	LYS-5
. .	. .	. .

### Visual analysis

**FIGURE 8:**
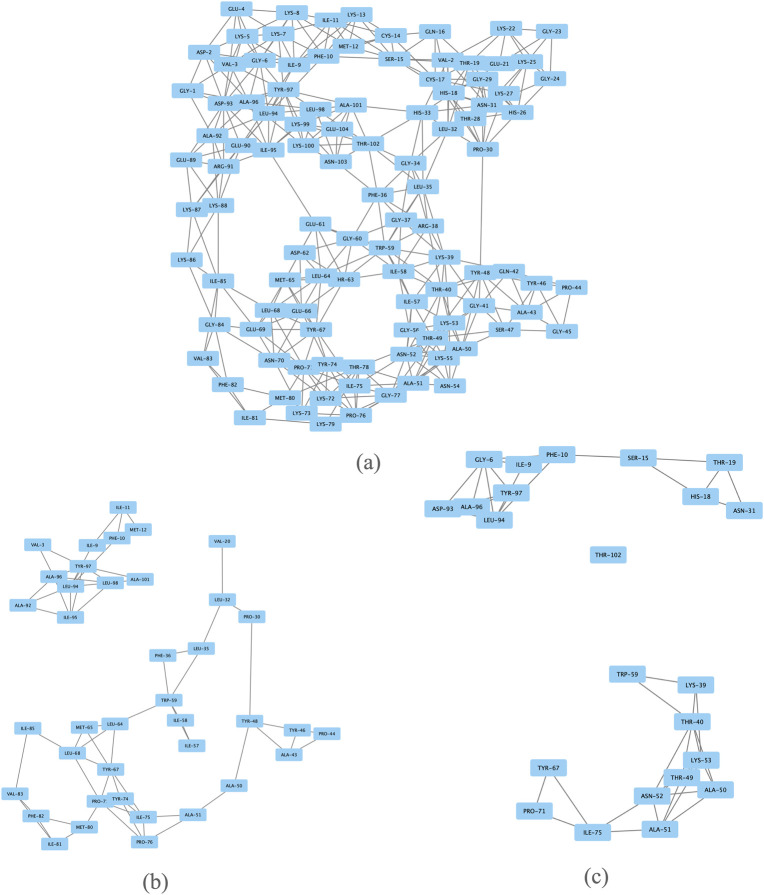
Cytoscape visualization based on SIF import. **(a)** Entire protein contact network generated from the SIF file; **(b)** Isolation of hydrophobic residues forming the protein core; **(c)** High-degree nodal hubs (top 10%) emphasizing critical functions.

The SIF-based visualization in Cytoscape (Figure 8) provides independent validation of the network topology derived from the application. The complete network view illustrates the overall protein nodal network (Figure 8a). Filtering for specific physicochemical properties clearly isolates the dense hydrophobic core scaffold buried within the protein interior (Figure 8b). Furthermore, highlighting the top 10% high-degree nodes identifies critical structural anchors distributed throughout the tertiary fold (Figure 8c).

## Discussion

To evaluate the validity of PCNE beyond the Cytochrome c case study, the tool was used to analyse a diverse set of proteins including Collage, Keratin, Myosin, Amylase, Hemoglobin, Insulin, Ferritin, and Immunoglobin G. Hub residues were also identified using the tool and were compared against functional residues as reported in literature (Table 5), and their agreement was quantified using the Jaccard Index, J which is given by

$$J=\frac{\left|A\cap B\right|}{\left|A\cup B\right|}$$

**TABLE 5:** Validation of PCNE hub analysis.

Protein	PDB ID	Known functional residues	PCNE confirmed hubs	Novel predicted hubs	J	Ref
Collagen	1BKV	Gly (every 3rd) Pro/hyp, lys/hyl	Gly-12, Gly-15 Ala-13, Arg-14 Leu-16	N/A	1.00	Kramer et al., 1999
Keratin	6IUL	Leu/Ile (heptad a,d) Glu/Lys (e,g), cys	Cys-284*, Leu-88 Ile-134, Leu-48 Leu-124, Ile-140	Trp-80; Arg-87; Arg-129	0.54	Bunick and Mistone, 2017
Myosin	1MMG	Lys-185, Asn-233 Gly-240, Arg-245 Glu-459	Gly-240* (switch ii) Lys-185*	Cys-655; Gly-179 Gln-246	0.29	Gulick et al., 1997
Amylase	1HNY	Asp-197, Glu-233 Asp-300, Arg-195, Asn-298, Arg-337, His-201 His-305	Glu-233*; Arg-195* Asp-197*	Tyr-94; His-491	0.30	Brayer et al., 1995
Haemoglobin	1A3N	His-93 (proximal) His-63 (distal) Val-98 (β), Asp-94 His-146	His-87* (α-proximal) Gly-59 (distal his neighbor); Gly-64 (Beta-chain)	Val-62 (βchain-, bc = 0.114)	0.33	Tame and Vallone, 2000
Insulin	1ZNJ	Phe-24/25, Tyr-26 (b), His-10 (b), Gly-8, Cys-6/7/11/19	Cys-6*; Cys-11* Gly-8*; Cys-19*; Tyr-16	Val-12 (bc = 0.368); Tyr-19	0.44	Chang et al., 1997
Ferritin	1FHA	Glu-27, Glu-62 His-65 (ferroxidase), Tyr-34, Asp-131 Glu-134 (channel)	Glu-27*; Glu-62* Tyr-34*	Val-110 (bc = 0.131); Glu-107	0.38	Lawson et al., 1991
Immunoglobulin G1	1IGY	Trp/Tyr (cdr) Cys-220/226/229, Trp-36, Pro-44	Tyr-173* (bc = 0.239); Trp-35*; Tyr-36*; cys-23/88/134/194**	Phe-139 (bc = 0.155); Leu-136 (bc = 0.153)	0.50	Harris et al., 1998

* Recovered across ≥2 node representations; ** Local PDB, chain numbering.

bc = betweenness centrality.

Where A is the set of PCNE hubs and B is the set of known functional residues.

## Novel predicted hubs

Myosin: CYS-655 and GLY-179. In the case of myosin, we have CYS-655 and GLY-179. The former is a stand-out in the myosin Cα network as the only hub with the highest degree, 15. This places it in the SH2-reactive thiol region of *Dictyostelium* myosin II, a structure essential for coupling the mechanical lever arm to the catalytic domain (Rayment et al., 1993). That we could recover CYS-655 without having to type any chemical interactions tells us that spatial proximity is all you need to localize such a mechanically vital spot. Then there is GLY-179, which shows a high betweenness centrality right next to the relay helix, where mechanochemical force is transmitted. One would expect to find glycines at these hinge points in the relay; they are conserved in myosin of all classes (Rayment et al., 1993).

Amylase: TYR-94 and HIS-491. TYR-94 was the highest-degree hub in the Cβ analysis (degree = 13), positioned at the substrate-binding cleft entrance of human pancreatic α-amylase, consistent with a structural gating function documented in crystallographic studies (Brayer et al., 1995). HIS-491 was recovered consistently across Cα and Cβ analyses in proximity to the calcium coordination site, its topological presence suggests a contribution to active site geometry maintenance (Brayer et al., 1995).

Hemoglobin: VAL-62 (β-chain). VAL-62 yielded the highest betweenness centrality in the hemoglobin SC-centroid analysis (BC = 0.1144), occupying a position topologically equivalent to the documented steric gatekeeper VAL-98. Its identification across representations as the primary betweenness bottleneck independently supports its classification as a haem-pocket structural control point (Tame and Vallone, 2000).

Ferritin: VAL-110. VAL-110 had the highest betweenness centrality in the ferritin SC-centroid analysis (BC = 0.1314) with no currently annotated functional role. Structurally, it sits at the junction of two α-helical bundle subunits, suggesting a conformational relay function between subunit interfaces. This, we believe, is a novel prediction from this analysis, and we suggest targeted mutational studies to assess its role in subunit assembly or iron-channelling dynamics (Lawson et al., 1991).

Immunoglobulin G: TYR-173. TYR-173 had the highest betweenness centrality across the entire multi-protein dataset (BC = 0.2394), approximately three times that of the next most central residue. Located at the variable constant (VC) domain interface, this value is consistent with a global bottleneck function aiding the conformational coupling between the antigen-binding and effector domains (Harris et al., 1998).

Insulin: VAL-12 (β-chain). VAL-12 in the SC-centroid analysis produced the highest betweenness centrality in the insulin dataset (BC = 0.368), flanked by LEU-11 and LEU-15, forming the internal packing scaffold of the B-chain helix. Its high degree of betweenness in the SC-centroid representation where side-chain chemical mass is incorporated into distance calculations might suggest it is critical for maintaining the compact hydrophobic core that stabilizes the insulin monomer prior to receptor engagement (Chang et al., 1997).

## Limitations and future work

Despite a few advancements, our current tool still has areas that remain basic. Although multiple residue representations including Cα atoms, Cβ atoms and side-chain residues are supported for the construction of protein contact networks, this application uses a geometric distance-based cutoff, and does not explicitly characterize physiochemical interactions such as hydrogen bonds, hydrophobic interactions, salt bridges or *π*-*π* stacking. Tools such as COCOMAPS 2.0 (Chawla et al., 2025) provide detailed analyses of protein-protein interfaces, including buried surface area calculations and characterization of non-covalent interactions within subunits of multimeric proteins. While these features fall outside the current scope of PCNE, integrating them in the future could enhance the biological interpretability of observations based on protein networks.

Consequently, the networks generated are coarse-grained abstractions of protein structures rather than fully atomistic interaction models. In comparison, tools like RING (Piovesan et al., 2016) provide more detailed interaction-specific characterization. Additionally, ProSNEx’s (Aydınkal et al., 2019) integration of ConSurf conservation scores and NAPS’s (Chak et al., 2016; Chakrabarty et al., 2019) spectral analysis capabilities offer valuable layers of biological context not yet included in this tool.

In addition, currently the tool does not perform automated *in silico* mutational or perturbation analysis internally. However, mutated protein structures can be generated using molecular modeling platforms such as PyMol and then uploaded and analysed within the framework of our tool to evaluate changes in the network topology, centrality and community organization. Future modifications will focus on integrating interaction-specific contact characterization, evolutionary and energetic metrics, and automated mutational analysis workflows to further improve the biological relevance of the topological insights.

## Conclusion

Protein contact network (PCN) platforms that have been established feature robust capabilities, ranging from data integration to interaction typing at the atomic level. However, they are often used in fragmented workspaces that separate data processing from structural visualization. Most current tools require web server access or rely heavily on external software like Cytoscape to deliver comprehensive insights. The application developed in this study bridges this gap by offering a framework that combines structural biology with network science, thereby removing the conflict between data computation and structural interpretation and enabling real-time analysis. Unlike standard web servers, which may utilize job queues or background processing, this application adopts a session-based approach. Its distinctive feature is a multi-dimensional visualization method. Unlike other tools that provide only abstract visualisation, this tool integrates interactive 3D spatial network visualisation with a quantitative matrix heatmap and statistical scatter plots. This allows users to directly connect spatial folding patterns with topological values and contact density maps within a single environment. Furthermore, this tool bridges the gap between biological data and topological abstraction by providing automatic physicochemical classification. Residues are colour-coded (Hydrophobic, Acidic, Basic, Polar) in the 3D network, highlighting the physicochemical properties underlying structural hotspots—an analysis typically requiring manual selection or filtering in other tools. The Wasserman-Faust normalisation of closeness centrality calculation ensures mathematical accuracy when analysing disconnected protein chains, a detail often not explicitly mentioned in other software. Additionally, the inclusion of an SIF export function makes this tool a valuable pre-processor for integration with Cytoscape software for advanced system-level analyses.

## Data Availability

The original contributions presented in the study are included in the article/Supplementary Material, further inquiries can be directed to the corresponding author.
